# A novel clinical prediction scoring system of high-altitude pulmonary hypertension

**DOI:** 10.3389/fcvm.2023.1290895

**Published:** 2024-01-08

**Authors:** Yanxi Zeng, Gulinigeer Zhakeer, Bingyu Li, Qing Yu, Mingyuan Niu, Nuerbiyemu Maimaitiaili, Ma Mi, Zhuoga Deji, Jianhui Zhuang, Wenhui Peng

**Affiliations:** ^1^ Department of Cardiology, Shigatse People’s Hospital, Tibet, China; ^2^ Department of Cardiology, Shanghai Tenth People’s Hospital, School of Medicine, Tongji University, Shanghai, China

**Keywords:** high-altitude pulmonary hypertension, high-altitude, GENTH score model, prediction model, pulmonary hypertension

## Abstract

**Background:**

High-altitude pulmonary hypertension (HAPH) is a common disease in regions of high altitude where performing right heart catheterization (RHC) is challenging. The development of a diagnostic scoring system is crucial for effective disease screening.

**Methods:**

A total of 148 individuals were included in a retrospective analysis, and an additional 42 residents were prospectively enrolled. We conducted a multivariable analysis to identify independent predictors of HAPH. Subsequently, we devised a prediction score based on the retrospective training set to anticipate the occurrence and severity of HAPH. This scoring system was further subjected to validation in the prospective cohort, in which all participants underwent RHC.

**Results:**

This scoring system, referred to as the **GENTH** score model (***G***lycated hemoglobin [OR = 4.5], ***E***chocardiography sign [OR = 9.1], ***N***ew York Heart Association-functional class [OR = 12.5], ***T***otal bilirubin [OR = 3.3], and ***H***ematocrit [OR = 3.6]), incorporated five independent risk factors and demonstrated strong predictive accuracy. In the training set, the area under the curve (AUC) values for predicting the occurrence and severity of HAPH were 0.851 and 0.832, respectively, while in the validation set, they were 0.841 and 0.893. In the validation set, GENTH score model cutoff values of ≤18 or >18 points were established for excluding or confirming HAPH, and a threshold of >30 points indicated severe HAPH.

**Conclusions:**

The GENTH score model, combining laboratory and echocardiography indicators, represents an effective tool for distinguishing potential HAPH patients and identifying those with severe HAPH. This scoring system improves the clinical screening of HAPH diseases and offers valuable insights into disease diagnosis and management.

## Introduction

Pulmonary hypertension (PH) is a multifaceted pathophysiological disorder associated with a spectrum of clinical conditions. It is defined as mean pulmonary arterial pressure (mPAP) >20 mmHg at rest, as determined through right heart catheterization (RHC) (1). The primary underlying pathophysiological process of PH is pulmonary vascular remodeling (2), a phenomenon that can ultimately result in right heart failure (3).

High-altitude pulmonary hypertension (HAPH) is classified as group 3 PH, primarily attributed to prolonged exposure to high-altitude environments (4, 5). These high-altitude regions are typically 2,500 m above sea level (6), inhabited by over 140 million permanent residents, with an additional 40 million temporary visitors (7). The living conditions between low-altitude plains (<1,000 m) and high-altitude areas (>2,500 m) (8) differ significantly, giving rise to various health challenges. The notable factors affecting the health of individuals include hypoxia and hypobaric conditions, resulting in hypoxemia, symptomatic excessive erythrocytosis, and elevated mPAP (9). The diagnostic criteria for HAPH typically involve mPAP measurements obtained through right heart catheterization (mPAP_RHC_), with mPAP_RHC_ >30 mmHg, as defined by the Qinghai criteria established by the International Society for Mountain Medicine (ISMM) in 2005 (5, 10). However, owing to limited equipment and technological resources, RHC, the gold standard for HAPH diagnosis, is rarely conducted in high-altitude regions (5). Consequently, the diagnosis of HAPH often relies on imaging techniques, particularly echocardiography, which may be imprecise in predicting mPAP compared with RHC due to the high variability inherent in the spectrum of right heart dysfunction (11) and the potential impact of observer-dependent factors (12).

Previous research has confirmed that HAPH patients also often have polycythemia and hypoxemia, as well as structural and functional alterations in the right heart (13). However, the precise independent risk factors remain undetermined, and a comprehensive scoring system to aid in diagnosis has yet to be devised. Therefore, there is an urgent requirement to develop a dedicated scoring system that amalgamates clinical characteristics with echocardiography data to improve the early diagnosis and management of HAPH in high-altitude regions.

## Methods

### Study design and population

The study received ethical approval from the Shigatse People's Hospital (altitude 4,040 m) (14) institutional review board, and informed consent was obtained from all enrolled patients. As ethnicity influences the responses to chronic hypoxia exposure, the study was limited to Tibetans and excluded other ethnicities and residents who had migrated to the region. Blood samples were consistently collected at 7 am in a fasting state. In the retrospective training set, the inclusion criteria were as follows: (1) male and females aged 18–85; (2) admission to Shigatse People's Hospital between August 2020 and August 2022; (3) a documented history of residing in high-altitude areas for more than 20 years; (4) an initial diagnosis suggesting PH; and (5) performance of CTA during hospitalization. The exclusion criteria for the retrospective training set included: (1) individuals with contraindications for undergoing CTA or indications of chronic thromboembolic disease in CTA; (2) PaO_2_ of ≥83 mmHg; (3) a documented history of left heart disease or echocardiography signs thereof, which include diastolic dysfunction, systolic dysfunction, and valvular diseases; (4) excessive erythrocytosis (defined as Hb of ≥19 g/dl for females and ≥21 g/dl for males); (5) the presence of severe hepatic or renal insufficiency; (6) pregnancy (for a detailed process of patient selection, please refer to Supplementary Figure 1).

For the prospective cohort, the inclusion criteria were further expanded as follows: (1) individuals admitted to Shigatse People's Hospital between August 2022 and August 2023; (2) no contraindications for undergoing RHC; and (3) provision of informed consent. Furthermore, echocardiography, CTA, and RHC (prospective cohort) were performed before medical therapy within 1 week after the first fasting blood. The chronological sequence of echocardiography, CTA, and RHC, along with the detailed process of patient selection, is presented in Supplementary Figure 2. Body surface area (BSA) and oxygen consumption (VO_2_) were estimated using established formulas (15). As per the Qinghai criteria, individuals were diagnosed with HAPH when mPAP was >30 mmHg (10). As patients with an mPAP >60 mmHg had a higher 30-day mortality rate (16), moderate and severe HAPH were defined as an mPAP of 30–60 mmHg and >60 mmHg, respectively (17, 18).

### Echocardiography

Transthoracic echocardiography was conducted in accordance with the guidelines provided by the American Society of Echocardiography (19). The following parameters were measured: vertical and transverse diameters of the left and right atrium and ventricle, main pulmonary artery diameter, and left ventricle ejection fraction (LVEF) (19). It is worth mentioning that the parameters of the right atrium and ventricle were measured using a right ventricle-focused apical four-chamber view (A4C), which was based on the A4C by adjusting the transducer to maximize the right ventricle visualized area. The main pulmonary artery diameter was measured using a pulmonic valve (PV)-focused parasternal short-axis (PSAX) view (20). To estimate the pressure gradient between the right atrium and right ventricle, the modified Bernoulli equation was applied, relying on the tricuspid regurgitation pressure gradient (TRPG) (21). Additionally, the right atrial pressure was evaluated and, in combination with TRPG, used to estimate pulmonary arterial systolic pressure (PASP) (22).

### Computed tomography angiography (CTA) acquisition

CTA scans were acquired using United Imaging scanners, with patients in a supine position and instructed to hold their breath during inspiration. The imaging parameters were set as follows: a tube voltage of 120 kVp, tube current of 300 mA, tube rotation time of 0.3 s, and collimator width of 64 mm × 0.625 mm. An iodinated contrast agent (Lomeprol, Bracco Sine, China, 400 mg/ml) was intravenously administered via a double-syringe power injector through the median cubital vein. The intelligent tracking mode was employed, and a 60 ml contrast agent was infused at a rate of 4 ml/s. When the density of the main pulmonary artery reached the predefined peak of 80 Hounsfield Units (HU), the scanning was triggered (23). On the four-chambered transverse view, measurements were taken for the atrial diameter, left lower bronchus, and arterial diameter. The left lower artery-bronchus ratio (ABR) was computed at the junction of the left main bronchus. The left lower ABR and the ratio of right to left atrial diameter (rRLA) were integrated into the prediction model described in previous publications. The model for calculating mPAP_Predicted_ was as follows: mPAP_predicted_ = −34 + 40 × left lower ABR + 7 × rRLA (*r* = 0.907, *R*^2^ = 0.823, *p* < 0.0001). This model demonstrated a strong fit and served as a reliable alternative to RHC for assessing mPAP (24, 25).

### Right heart catheterization and pulmonary angiography

In the prospective validation cohort, RHC was conducted following standard procedures by an interventional cardiologist with over 10 years of experience (1). Using local anesthesia, an introducer sheath was positioned in the right femoral vein, and a JR 4.0 catheter was advanced into the pulmonary artery to measure mPAP (mPAP_RHC_). mPAP_RHC_ was recorded in the main pulmonary artery, just above the pulmonary valve. Subsequently, systolic and diastolic pressures of the right ventricle, right atrium, and superior vena cava were measured through standard image analysis techniques (26). To enhance the visualization of the pulmonary vessels. a contrast medium was injected into the openings of the left and right upper and lower pulmonary arteries (26).

### Risk factor screening and transformation

Following difference and correlation analyses, the parameters transformed dichotomous variables and then incorporated them into a multivariate logistics regression. The upper and lower limits for these dichotomous variables were determined based on the calibration range of the laboratory instruments at Shigatse People's Hospital (27, 28), as well as the criteria outlined in the guidelines for hyperbilirubinemia (29), hyperuricemia (30), and diabetes (31). In line with recent updates to guidelines, in which TRPG and PASP from echocardiography have been replaced by other relevant echocardiographic signs that contribute to the assessment of the probability of PH (1, 32, 33). We defined echocardiographic signs indicative of right heart dysfunction in this study as follows: (1) vertical and transverse enlargement of the right atrium; (2) vertical and transverse enlargement of the right ventricle; and (3) dilation of the main pulmonary artery. These three indicators were collectively referred to as “Echocardiography signs” within the GENTH score model. A prediction score system, termed the GENTH score model, was devised to predict the occurrence and severity of HAPH. The GENTH score model comprises five critical risk factors: ***G***lycated hemoglobin, ***E***chocardiography sign, ***N***ew York Heart Association-functional class (NYHA-FC), ***T***otal bilirubin, and ***H***ematocrit.

### Statistical analysis

Characteristics of the training and validation patients were described by mPAP_Predicted_ and mPAP_RHC_, respectively. The normal distribution of measurements was assessed using Kolmogorov–Smirnov's test and Shapiro–Wilk's test. Normally distributed data, non-normally distributed data, and categorical data are presented as mean ± standard deviation, median (the first quantile and the third quantile), and frequency, respectively. Group differences were analyzed as appropriate using one-way ANOVA, a Kruskal–Wallis *H*-test, or chi-square test. The trends in continuous variables or rates of dichotomous variables were assessed through linear regression analysis and Cochran-Armitage, respectively. Lasso regression was used to screen for the risk factors. Spearman's correlation was employed to examine the relationship between parameters and mPAP_Predicted_. Univariate and multivariate logistic regression analyses were conducted to calculate odds ratios (ORs) and 95% confidence intervals (CIs). Variables that remained statistically significant after the multivariate logistic analysis were considered candidates for the development of a novel scoring model, and the relevant points were determined based on the OR values, which reflect their impact on disease diagnosis. The receiver operating characteristic (ROC) curve was generated, and the area under the ROC curve (AUC) was calculated to evaluate the predictive performances of the scoring model. A nomogram was used to visualize the scoring model based on multivariate logistic regression analysis. The C-index was calculated to assess the predictive performances of the scoring models. Cutoff values were calculated to achieve a specificity of at least 95%. All statistical analyses were performed using R v.4.1.2 (R Foundation for Statistical Computing, Vienna, Austria) and SPSS v.26.0 (SPSS Inc. Chicago, Illinois, USA). A two-sided *p* < 0.05 was considered statistically significant.

## Results

### Baseline characteristics of individuals in the training set

In the training group, all participants underwent pulmonary CTA to gather data on rRLA and left lower ABR, enabling the calculation of mPAP_Predicted_. The average mPAP_Predicted_ was 47 mmHg, ranging from 14 to 113 mmHg. There were no significant differences among the groups with respect to gender, age, or living altitude. However, patients with HAPH were more likely to experience cardiac dysfunction and had higher NYHA-FC grades. Laboratory tests revealed that HAPH patients often exhibited polycythemia, as indicated by elevated levels of erythrocytes, hemoglobin, and hematocrit (*p* for trend <0.05). In comparison with healthy highlanders (HH), HAPH patients showed elevated transaminase, uric acid, and total bilirubin, and a prolonged prothrombin time (*p* for trend <0.05). Blood gas analysis unveiled that HAPH patients frequently had metabolic alkalosis, characterized by increased PaCO_2_ and base excess. Moreover, HAPH patients had lower total cholesterol and triglyceride levels, and higher glycated hemoglobin levels, implying the influence of hypoxia and the low atmospheric pressure environment on lipid and glucose metabolism in high-altitude regions (Table 1). Notably, there were no significant differences among the groups in terms of vital signs, height, weight, and metabolic indicators such as BMI, BSA, and VO_2_ as well as myocardial enzymes (Supplementary Table 1). Additional information regarding the between-group differences and 95% CIs for clinical characteristics is provided in Supplementary Table 2.

**Table 1:** Baseline characteristics of individuals in the retrospective group.

Characteristic	Total	3-level of predicted mPAP			*p* value	*p* for trend
		≤30 (*n* = 25)	30–60 (*n* = 92)	>60 (*n* = 31)		
Gender, male (%)	77 (52)	16 (20.8)	43 (55.8)	18 (23.4)	2.919	0.232
Age, years	64 (51, 73)	57 (37, 71)	64 (52, 73)	65 (56, 74)	0.304	0.215
Altitude, m	4,100 (4,000, 4,200)	4,100 (4,000, 4,300)	4,000 (3,975, 4,200)	4,100 (4,000, 4,300)	0.111	0.871
NYHA-FC						
Grade 1 (%)	21 (14.2)	7 (33.3)	14 (66.7)	0 (0)		
Grade 2 (%)	32 (21.6)	8 (25.0)	22 (68.8)	2 (6.2)		
Grade 3 (%)	72 (48.6)	8 (11.1)	44 (61.1)	20 (27.8)		
Grade 4 (%)	23 (15.5)	2 (8.7)	12 (52.2)	9 (39.1)	0.001^a^	0.001^b^
Erythrocyte, ×10^12^/L	4.8 (4.0, 5.5)	4.4 (3.6, 5.2)	4.8 (4.0, 5.3)	5.2 (4.0, 5.9)	0.038^a^	0.003^b^
HGB, g/L	136 (116, 164)	118 (103, 142)	137 (115, 159)	147 (128, 171)	0.005^a^	0.007^b^
Hematocrit, %	44 (37, 49)	36 (34, 40)	44 (37, 48)	49 (45, 59)	<0.001^a^	<0.001^b^
ALT, U/L	33 (18, 61)	46 (27, 68)	29 (17, 57)	41 (19, 67)	0.209	0.257
AST, U/L	34 (24, 58)	53 (26, 81)	31 (23, 51)	46 (27, 65)	0.028^a^	0.486
Albumin, g/L	37 (33, 42)	40 (35, 43)	38 (32, 42)	36 (32, 39)	0.086	0.350
Total bilirubin, μmol/L	20 (12, 34)	21 (14, 27)	17 (10, 33)	33 (20, 47)	<0.001^a^	0.005^b^
CREA, μmol/L	82 (66,100)	91 (73, 100)	80 (61, 97)	86 (73, 106)	0.235	0.096
UA, μmol/L	351 (252, 485)	309 (222, 434)	344 (248, 451)	442 (322, 656)	0.006^a^	0.025^b^
BUN, mmol/L	4.0 (2.9, 6.0)	4.5 (3.3, 5.5)	3.8 (2.7, 5.4)	5.2 (3.2, 7.3)	0.094	0.436
PT,s	13 (12, 15)	13 (11, 14)	13 (12, 15)	15 (13, 17)	0.027^a^	0.010^b^
PaO_2_, mmHg	47 (42, 57)	49 (39, 62)	47 (42, 56)	45 (42, 58)	0.822	0.397
PaCO_2_, mmHg	33 (28, 39)	29 (23, 35)	32 (30, 39)	37 (27, 43)	0.046^a^	0.013^b^
BE, mmol/L	−0.5 (−4.0, 2.0)	−3.0 (−4.5, −0.2)	0.0 (−4.0, 1.8)	0.0 (−3.0, 3.4)	0.036^a^	0.046^b^
HCO_3_^−^, mmol/L	23 (21, 26)	22 (18, 24)	24 (21, 27)	23 (21, 28)	0.157	0.171
TC, mmol/L	3.0 (2.5, 3.4)	3.2 (2.5, 3.5)	3.1 (2.7, 3.5)	2.5 (2.3, 3.3)	0.048^a^	0.086
TG, mmol/L	0.99 (0.86, 1.04)	1.04 (0.95, 1.10)	0.99 (0.80, 1.03)	0.88 (0.70, 1.13)	0.025^a^	0.261
Blood glucose, mmol/L	4.5 (4.0, 5.1)	4.7 (4.3, 5.3)	4.5 (4.0, 5.0)	4.5 (3.9, 4.7)	0.120	0.626
Glycated hemoglobin, %	5.91 (5.51, 6.20)	5.51 (5.50, 5.86)	5.80 (5.40, 6.16)	6.24 (6.00, 6.50)	<0.001^a^	0.001^b^

Data are mean ± SD or median (P_25_, P_75_). Group differences were assessed by one-way ANOVA, a chi-square test, or Kruskal–Wallis H tests. ALT, alanine aminotransferase; AST, aspartate aminotransferase; BE, base excess; BUN, blood urea nitrogen; CREA, creatinine; HCO_3_^−^, bicarbonate concentration; HGB, hemoglobin; NYHC-FC, New York Heart Association-functional class; PaCO_2_, carbon dioxide partial pressure; PaO_2_, oxygen partial pressure; PT, prothrombin time; RBC, erythrocyte; TC, total cholesterol; TG, triglyceride; UA, uric acid.

^a^ *p *< 0.05, the group difference assessed by the Kruskal–Wallis H test or a chi-square test was significant.

^b^ *p* < 0.05, the trend evaluated by linear regression analysis or a Cochran–Armitage test was significant.

### Echocardiography characteristics in the training set

**Figure 1:**
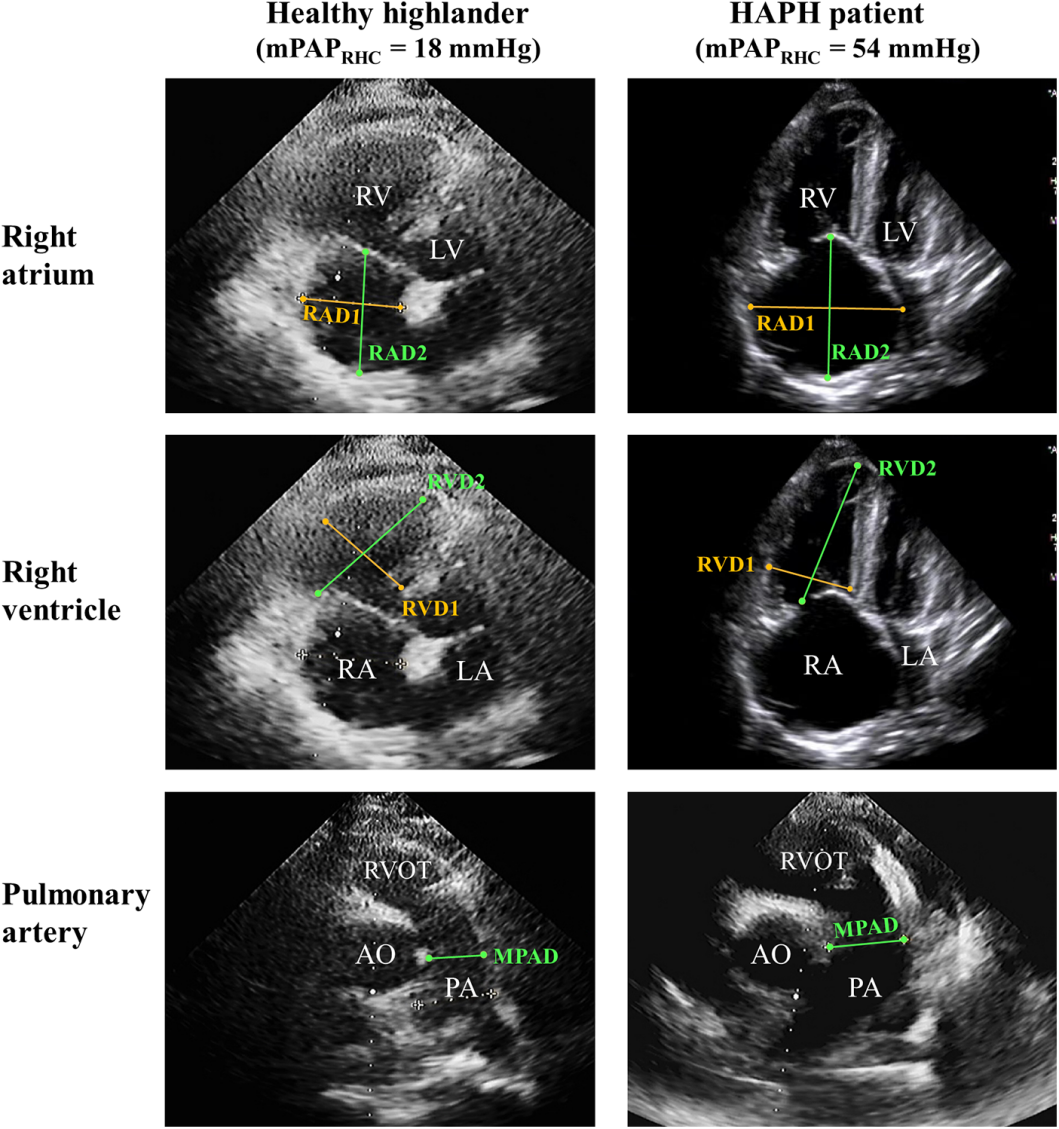
Representative imaging of the echocardiographic sign of right heart dysfunction. Right ventricle-focused apical four-chamber view and pulmonic valve-focused parasternal short-axis view of HH and HAPH patients. PA, pulmonary artery; RAD1, transverse diameter of right atrium; RAD2, vertical diameter of right atrium; RVD1, transverse diameter of right ventricle; RVD2, vertical diameter of right ventricle.

The representative imaging for parameters of the right atrium and ventricle is shown in Figure 1. Patients with abnormal mPAP_Predicted_ were more likely to exhibit several echocardiographic abnormalities, including an enlarged right atrium and ventricle in both transverse and vertical dimensions (*p* for trend <0.01), a dilated main pulmonary artery (*p* for trend <0.01), and an elevated TRPG and PASP (*p* for trend <0.05). However, there were no significant differences in left heart diameter and LVEF among the three groups with varying mPAP_Predicted_. These findings indicated that HAPH patients are more likely to display signs of right heart dysfunction, as evidenced by echocardiography (*p* for trend <0.05; illustrated in Table 2). Further details regarding the between-group differences and 95% CIs for echocardiography characteristics are provided in Supplementary Table 3.

**Table 2:** Comparisons of echographic parameters in the retrospective group.

Characteristic	Group	Total	3-level of predicted mPAP			*p* value	*p* for trend
			≤30 (*n* = 25)	30–60 (*n* = 92)	>60 (*n* = 31)		
RAD1, mm	Enlarge (%)	101 (68)	9 (36)	69 (75)	23 (74)	0.001^a^	0.005^b^
RAD2, mm	Enlarge (%)	90 (61)	6 (24)	60 (65)	24 (77)	<0.001^a^	0.001^b^
RVD1, mm	Enlarge (%)	91 (61)	6 (24)	60 (65)	25 (81)	<0.001^a^	0.001 ^b^
RVD2, mm	Enlarge (%)	39 (26)	1 (4)	26 (28)	12 (39)	0.011^a^	0.004^b^
LAD1, mm	Enlarge (%)	77 (52)	8 (32)	54 (59)	15 (48)	0.054	0.300
LAD2, mm	Enlarge (%)	35 (24)	2 (8)	25 (27)	8 (26)	0.128	0.150
LVEDD, mm	Enlarge (%)	20 (14)	2 (8)	11 (12)	7 (23)	0.221	0.102
LVESD, mm	Enlarge (%)	26 (18)	3 (12)	16 (17)	7 (23)	0.584	0.301
MPAD, mm	Enlarge (%)	91 (61)	5 (20)	63 (69)	23 (74)	<0.001^a^	0.001^b^
Sign of right heart dysfunction	0 or 1 (%)	52 (35)	19 (76)	26 (28)	7 (23)		
	≥2 (%)	96 (65)	6 (24)	66 (72)	24 (77)	<0.001^a^	<0.001^b^
TRPG, mmHg		32 (24, 46)	25 (8, 34)	32 (25, 50)	35 (25, 53)	0.009^a^	0.014^b^
PASP, mmHg		41 (30, 54)	33 (23, 41)	42 (32, 58)	46 (30, 63)	0.009^a^	0.012^b^
LVEF, %		60 (48, 64)	55 (49, 64)	59 (47, 64)	61 (51, 65)	0.542	0.943

Data are mean ± SD or median (interquartile range), and the categorical variables are presented as absolute numbers (percentages). Group differences were assessed by one-way ANOVA, Fisher's exact test or Kruskal–Wallis H tests. LAD1, transverse diameter of left atrium; LAD2, vertical diameter of left atrium; LVEDD, left ventricle end-diastolic diameter; LVEF, left ventricular ejection fraction. LVESD, left ventricle end-systolic diameter; MPAD, main pulmonary artery diameter measured by echocardiography; PASP, echocardiographic pulmonary arterial systolic pressure estimate; RAD1, transverse diameter of the right atrium; RAD2, vertical diameter of right atrium; RVD1, transverse diameter of right ventricle; RVD2, vertical diameter of the right ventricle; TRPG, tricuspid regurgitation differential pressure.

^a^ *p *< 0.05, the group difference assessed by a Kruskal–Wallis H test or chi-square test was significant.

^b^ *p *< 0.05, the trend evaluated by linear regression analysis or a Cochran–Armitage test was significant.

### Independent risk factors associated with HAPH

**Figure 2:**
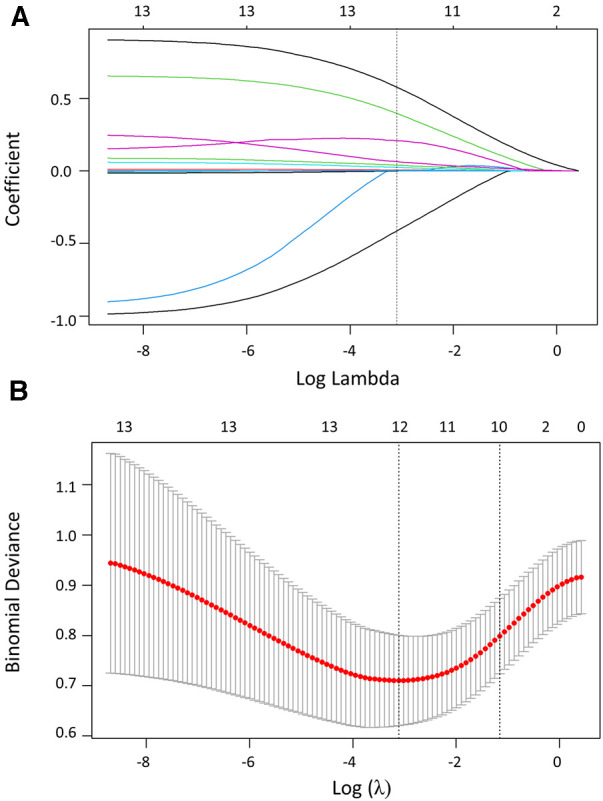
Variable screening based on lasso regression. (**A**) The variation characteristics of the coefficient of variables. (**B**) The selection process of the optimum value of the parameter log (*λ*) in the Lasso regression model using the cross-validation method.

The parameters, including basic characteristics and echocardiography signs, were considered for potential predictive variables. Lasso regression was used to screen parameters, and the 10-fold cross-validation method was applied to the iterative analysis (Figure 2). The screened variables included age, gender, NYHA-FC, hemoglobin, hematocrit, total bilirubin, uric acid, prothrombin time, echocardiography sign, glycated hemoglobin, PaCO_2_, and BE. Correlation analyses revealed positive correlations of mPAP_Predicted_ with NYHA-FC (*r* = 0.391, *p *< 0.001), hematocrit (*r* = 0.322, *p *< 0.001), uric acid (*r* = 0.323, *p *< 0.001), glycated hemoglobin (*r* = 0.500, *p *< 0.001), echocardiography sign (*r* = 0.411, *p *< 0.001), total bilirubin (*r* = 0.285, *p *< 0.001), and prothrombin time (*r* = 0.285, *p =* 0.001) (Table 3). Variables with a *p* ≤ 0.001 were transformed into dichotomous variables and subsequently compared between HH and HAPH, as well as between moderate and severe HAPH, respectively (Supplementary Table 4). These variables were then included in the subsequent logistic regression analysis.

**Table 3:** Spearman's correlation of mPAP_Predicted_ with potential predictors.

Parameter	M (P_25_, P_75_)	*r*	*p* value
mPAP_Predicted_	47 (35, 57)	/	/
NYHA-FC	3 (2,3)	0.391	<0.001^a^
RBC, ×10^12^/L	4.8 (4.0, 5.5)	0.202	0.014^a^
HGB, g/L	136 (116, 164)	0.229	0.005^a^
Hematocrit, %	44 (37, 49)	0.322	<0.001^a^
AST, U/L	34 (24, 58)	0.002	0.977
Total bilirubin, μmol/L	20 (12, 34)	0.285	<0.001^a^
Uric acid, μmol/L	351 (252, 485)	0.323	<0.001^a^
Prothrombin time, s	13 (12, 15)	0.259	0.001^a^
PaCO_2_, mmHg	33 (28, 39)	0.237	0.004^a^
BE, mmol/L	−0.5 (−4.0, 2.0)	0.230	0.006^a^
TC, mmol/L	3.0 (2.5, 3.4)	−0.110	0.181
TG, mmol/L	0.99 (0.86, 1.04)	−0.124	0.133
Glycated hemoglobin,%	5.91 (5.51, 6.20)	0.500	<0.001^a^
Echocardiography sign	3 (1, 3)	0.411	<0.001^a^
TRPG, mmHg	32 (24, 46)	0.274	0.009^a^
PASP, mmHg	41 (30, 54)	0.266	0.009^a^

Data are median (P_25_, P_75_). NYHC-FC, New York Heart Association-functional class; RBC, erythrocyte; AST; aspartate aminotransferase; PaCO_2_, carbon dioxide partial pressure; BE, base excess; TC, total cholesterol; TG, triglyceride.

^a^ *p *< 0.05, the Spearman's correlation coefficient was significant and the correlation between independent variables and mPAP_Predicted_ was significant.

### Construction of a novel prediction model of HAPH and severe HAPH

The results of the multivariate logistic regression analysis unveiled a set of independent risk factors associated with the diagnosis of HAPH compared with HH (*p* < 0.05). These factors included an NYHA-FC ≥ Grade III (OR: 4.1, *p *= 0.021), hematocrit of >45% (OR: 3.6, *p *= 0.045), glycated hemoglobin of >6% (OR: 4.5, *p *= 0.039), and an echocardiography sign of ≥2 points (OR: 9.1, *p *= 0.001). Further stratification of HAPH patients into moderate HAPH (30 mmHg < mPAP ≤ 60 mmHg) and severe HAPH (mPAP > 60 mmHg) identified additional independent risk factors for severe HAPH through multivariate logistic regression analysis, including an NYHA-FC ≥ Grade III (OR: 12.5, *p *= 0.004), hematocrit of >45% (OR: 3.2, *p *= 0.028), total bilirubin of >34 μmol/L (OR: 3.3, *p *= 0.037), and glycated hemoglobin of >6% (OR: 3.8, *p *= 0.012) (Table 4). The results of the univariate logistic regression are shown in Supplementary Table 5.

**Table 4:** Independent risk factors associated with HAPH and severe HAPH.

Parameter	HH vs. HAPH		Moderate vs. Severe HAPH	
	OR (95% CI)	*p* value	OR (95% CI)	*p* value
NYHA-FC				
Grade I or II	1	\	1	\
≥Grade III	4.091 (1.231–13.593)	0.021^a^	12.516 (2.281–68.692)	0.004^a^
Hematocrit, %				
≤45	1	\	1	\
>45	3.618 (1.031–12.692)	0.045^a^	3.239 (1.134–9.254)	0.028^a^
Total bilirubin, μmol/L				
≤34	1	\	1	\
>34	0.885 (0.190–4.125)	0.885	3.316 (1.077–10.207)	0.037^a^
Uric acid, μmol/L				
≤420	1	\	1	\
>420	0.541 (0.115–2.549)	0.437	0.822 (0.275–2.455)	0.822
Prothrombin time, s				
≤14	1	\	1	\
>14	2.173 (0.535–8.819)	0.277	1.940 (0.711–5.298)	0.196
Glycated hemoglobin,%				
≤6.0	1	\	1	\
>6.0	4.534 (1.078–19.068)	0.039^a^	3.771 (1.340–10.612)	0.012^a^
Echocardiography sign				
<2 points	1	\	1	\
≥2 points	9.059 (2.926–28.040)	0.001^a^	1.288 (0.401–4.143)	0.671

HH, healthy highlanders; NYHC-FC, New York Heart Association-functional class.

^a^ *p *< 0.05, the group difference assessed by the multivariate logistic regression test was significant.

To facilitate clinical application and assessment, a novel prediction model for HAPH diagnosis and severity classification, named the GENTH score model, was developed based on the OR values from multivariate logistic analysis. The GENTH scoring system incorporated the results of OR values in both multivariate logistics regressions, which could predict HAPH and its severity at the same time to improve convenience. Based on the independent predictors of HH vs. HAPH, the score of the highest proportional predictor (NYHA-FC) of moderate vs. severe HAPH was adjusted according to the OR = 12.5. Then, total bilirubin, the new predictor of moderate vs. severe HAPH was also added to the scoring system with its OR = 3.3. Each independent predictor of HAPH was assigned a weighted point value, with glycated hemoglobin receiving 5 points, echocardiography signs 9 points, NYHA-FC 13 points, total bilirubin 3 points, and hematocrit 4 points. This scoring system yielded a total score ranging from 0 to 34. A detailed explanation of the GENTH score model calculation is presented in Table 5.

**Table 5:** Calculation of the GENTH score.

Parameter	Points
Glycated hemoglobin, %	
≤6.0	0
>6.0	5
Echocardiography sign	
<2	0
≥2	9
NYHA-FC	
Grade I or II	0
≥Grade III	13
Total bilirubin, μmol/L	
≤34	0
>34	3
Hematocrit, %	
≤45	0
>45	4

NYHC-FC, New York Heart Association-functional class.

### The predictive and cutoff value of the GENTH score model

**Figure 3:**
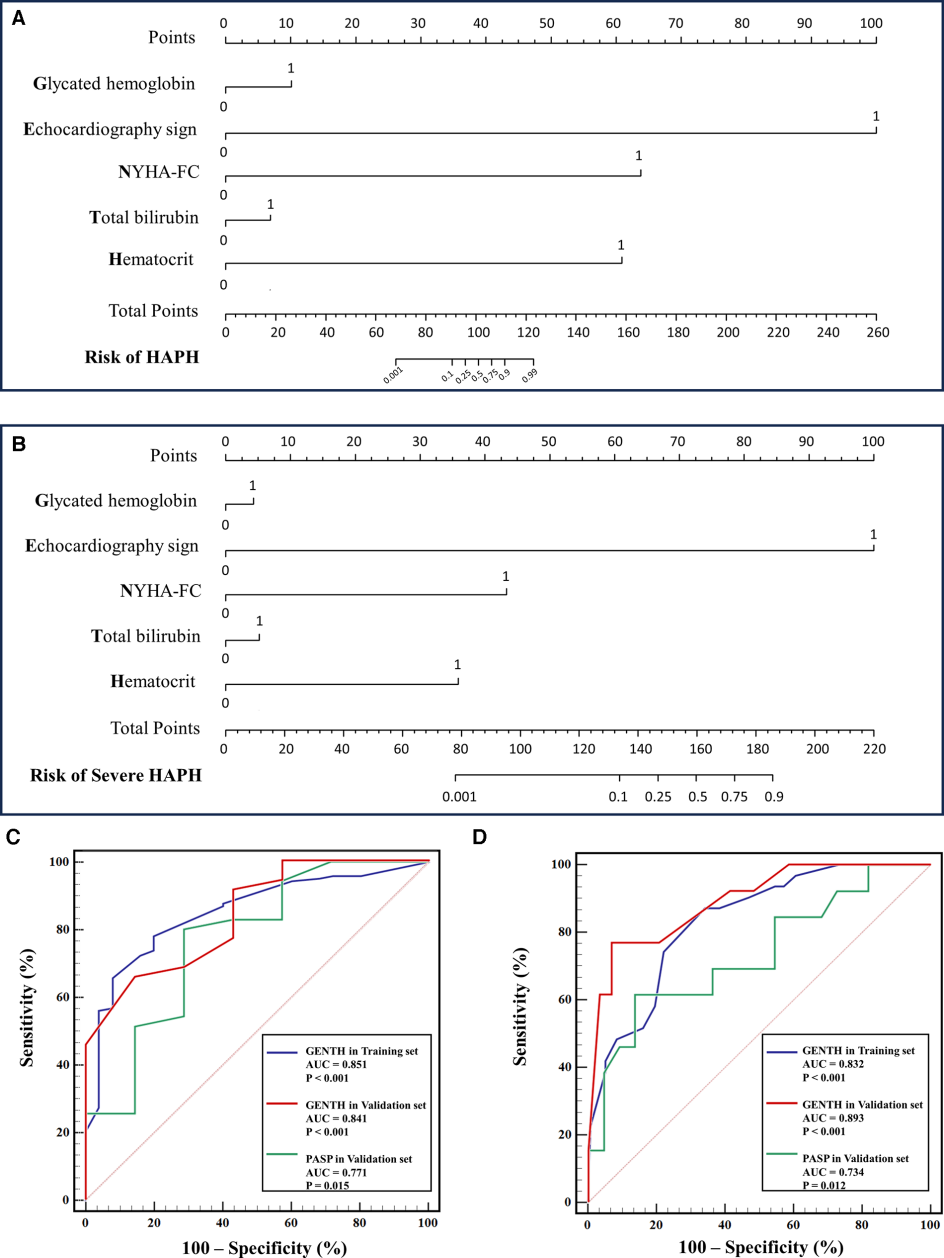
Nomogram and ROC curve of the GENTH score in the diagnosis and severity of HAPH. (**A**) Nomogram for the GENTH scoring system predicting HAPH in the validation set. (**B**) Nomogram for the GENTH scoring system predicting severe HAPH in the validation set. (**C**) ROC of the GENTH scoring system and PASP predicting HAPH in the training and validation sets. (**D**) ROC of the GENTH scoring system and PASP predicting severe HAPH in the training and validation sets.

The baseline characteristics and echocardiography sign of the validation set are presented in Supplementary Tables 6, 7. There was a high correlation between mPAP_RHC_ and mPAP_Predicted_ (*r* = 0.876, *p <* 0.001) (Supplementary Table 8). To facilitate the clinical service, the nomogram was used to visualize the GENTH scoring system, and the parameters of the model were derived from the results of multivariate logistic regression analysis as mentioned above (Figures 3A–B). The C-index of the GENTH score model for predicting HAPH was 0.878 (95% CI: 0.793–0.881) in internal validation and 0.959 (95% CI: 0.884–0.963) in external validation. As for predicting severe HAPH, the C-index was 0.853 (95% CI: 0.767–0.56) in internal validation and 0.914 (95% CI: 0.802–0.918) in external validation. To assess the predictive performance of the GENTH scoring model, ROC curve analysis was conducted, and AUC values were calculated to evaluate the consistency of mPAP and the GENTH score model when compared with echocardiography-derived PASP (Figures 3C–D). In the validation cohort, the AUC values for the GENTH score model in diagnosing HAPH and severe HAPH were 0.835 and 0.848, respectively. These results were higher than the AUCs obtained for echocardiography PASP, which were 0.771 for HAPH and 0.724 for severe HAPH. The GENTH score model cutoff values for diagnosing HAPH (mPAP of >30 mmHg) and severe HAPH (mPAP of >60 mmHg) with a specificity of ≥95% were determined to be >18 and >30 points, respectively. The corresponding sensitivity, specificity, positive predictive value (PPV), and negative predictive value (NPV) are presented in Table 6.

**Table 6:** Diagnostic accuracy of GENTH in the validation set.

	HAPH	Severe HAPH
Cutoff value	>18 points	>30 points
Sensitivity	45.7%	61.5%
Specificity	100%	96.6%
PPV	100%	88.9%
NPV	26.9%	84.8%
PLR	\	17.9
NLR	\	0.4

PPV, positive predictive value; NPV, negative predictive value; PLR, positive likelihood ratio; NLR, negative likelihood ratio.

**Figure 4:**
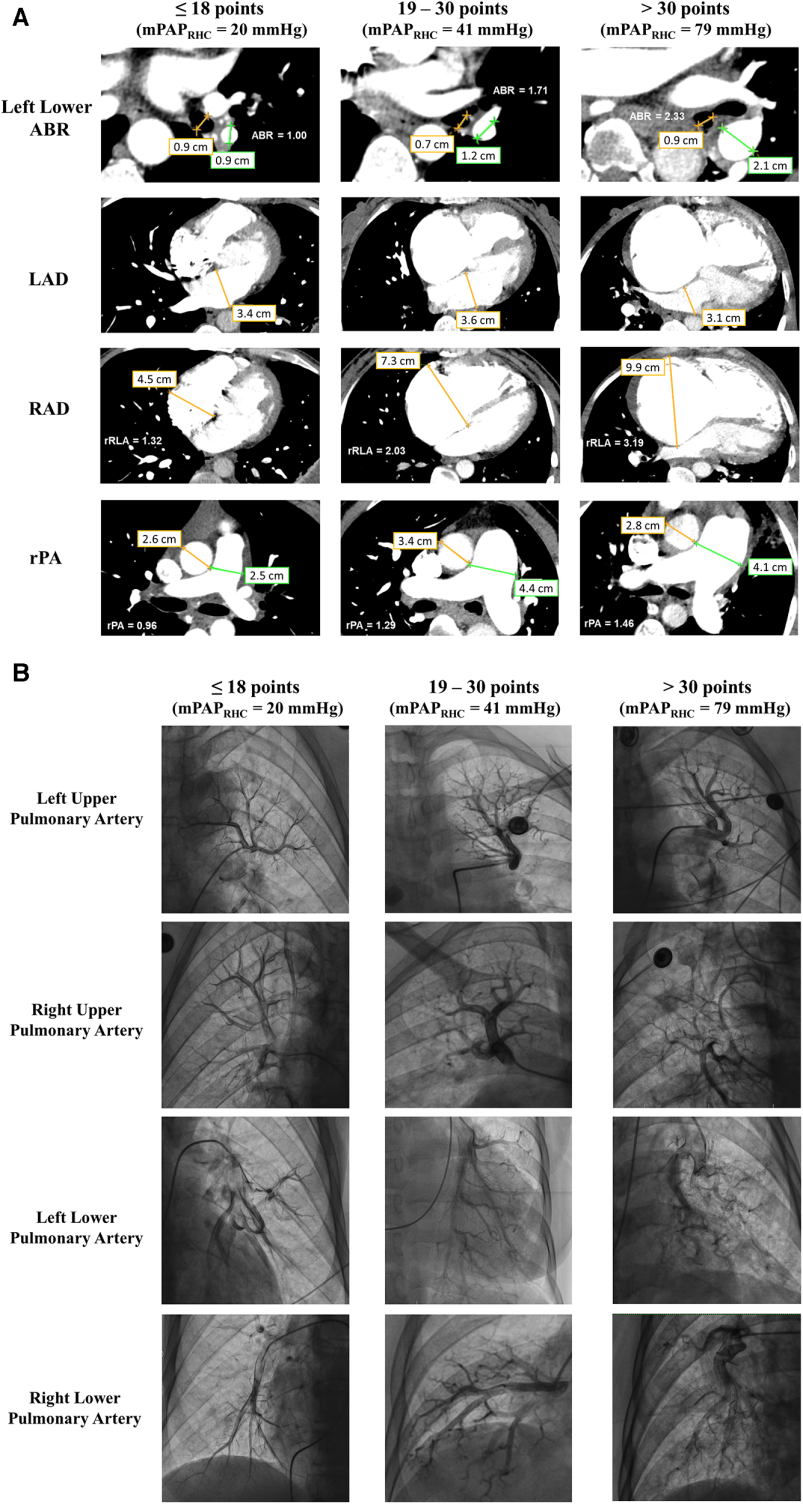
Imaging features of CTA and pulmonary arteriography in the GENTH score. (**A**) The diameter of the left lower pulmonary artery and bronchus, right and left atria, aorta, and main pulmonary artery in patients with different GENTH scores in the validation set. (**B**) Imaging features of pulmonary arteriography in patients with different GENTH scores in the validation set. ABR, pulmonary artery-bronchus ratio; LAD, left atrium diameter; RAD, right atrium diameter; rPA, ratio of the main pulmonary artery to aorta diameter; rRLA, ratio of the right to left atrial diameter.

Figure 4A illustrates the parameters from CTA in patients with different GENTH scores in the validation set. When compared with HH, HAPH patients were more likely to exhibit an enlarged right atrium, main pulmonary artery, and left pulmonary artery. Additionally, the ratios based on these parameters, such as rRLA, rPA, and left lower ABR, were increased. Subsequent RHC and pulmonary angiography were performed to confirm the diagnosis. In comparison with HH, HAPH patients with a GENTH score of >18 demonstrated enlarged, increased, and distorted arterial branches with rough intima, particularly noticeable in the lower pulmonary artery. This phenomenon was more pronounced in patients with a GENTH score of >30, which corresponds to higher mPAP values measured via RHC (Figure 4B).

## Discussion

Our study places particular emphasis on HAPH, a subset of PH that has generated relatively less global attention and faces challenges in terms of diagnosis and treatment, especially in high-altitude regions (5). We have introduced the GENTH score model, which is the first clinical prediction scoring system designed for both diagnosing HAPH and categorizing its severity. This scoring system has undergone validation in a prospective cohort, marking a significant step toward improved management of this condition.

The study commenced with a baseline analysis aimed at the early screening of risk factors in HAPH. Alterations in laboratory markers among HAPH patients include erythrocytosis and CO_2_ accumulation, which are likely linked to compensatory physiological responses developed because of prolonged chronic hypoxia (17). Moreover, compared with HH, HAPH patients exhibit variations in prothrombin time and metabolic indicators, reflecting disruptions in lipid and cholesterol homeostasis, macronutrient metabolism, and the coagulation function of the liver (34). These findings suggest that the metabolic changes observed in HAPH patients may influence liver function through three potential mechanisms. First, the hypoxia-inducible factor (HIF) family is activated during cellular adaptation to hypoxic stress (35). HIF-1 and HIF-2α are upregulated in hepatocytes under hypoxic conditions, impacting glucose metabolism (36) and leading to lipid accumulation within hepatocytes (37), respectively. Second, the liver plays a critical role in blood volume regulation (38), and it is not uncommon to observe hyperbilirubinemia in long-standing PH, possibly arising from subsequent liver congestion associated with polycythemia (39). Moreover, HAPH patients often develop right ventricular failure, which can lead to liver congestion (40) and subsequently elevate myocardial markers and transaminases (41). The metabolic changes in HAPH patients may be attributed to chronic hypoxia, an increase in blood volume, and right ventricular dysfunction. It is noteworthy that compared with HH, HAPH patients typically have lower total cholesterol and triglyceride levels. This difference can be linked to hepatocyte lipid accumulation (42) and alterations in peripheral lipid metabolism under hypoxic conditions (43). These factors may potentially contribute to the decreased incidence of obesity in high-altitude regions (4, 44). Moreover, the prevalence of pulmonary embolism in the retrospective cohort was high at 21% (52/248). Previous studies reported that high altitude was the risk factor for pulmonary embolism, and the potential mechanisms may be related to activated coagulation and increased inflammation under hypoxic stimulation (45). In addition, the prevalence of pulmonary embolism was higher in patients with polycythemia (39%) than in those without polycythemia (11%) (46). Therefore, this phenomenon may result from secondary polycythemia and hypoxia-activated coagulation and inflammation. Furthermore, as we excluded patients without CTA in this research, which was more commonly performed in patients with suspected pulmonary embolism, this ratio may be biased.

The selected predictors of the GENTH score model were closely associated with various aspects, including blood volume, cardiac function, cardiac structural changes, and metabolism. The treatment and prognosis of PH are closely related to the recovery of left or right heart function (47). Notably, right ventricular functional recovery (RVFnRec) has emerged as a novel endpoint in the successful treatment of pulmonary hypertension (48). This underscores the pivotal role played by echocardiography and cardiac function classification in the predictive accuracy of the GENTH score model. The echocardiography sign of right heart dysfunction was assigned with the highest points for HAPH, highlighting the importance of detecting right heart structural changes (13) as a potential early indicator for HAPH. In the case of severe HAPH, we often observe pulmonary vascular remodeling (49) and cardiac dysfunction (3), manifested through worsening clinical symptoms and elevated NYHA-FC grades. Therefore, echocardiography signs were excluded from the independent predictors, reflecting the compensatory and potentially reversible dilatation of the right heart and pulmonary artery in the early stages (50). During this phase, the proportion of NYHA-FC elevated. Moreover, previous studies have reported that the initial mPAP of HAPH patients tends to be higher than that of PH patients in low-altitude regions (17), despite the absence of pronounced clinical symptoms in the early stages (51). Therefore, it is important for clinicians to prioritize the early detection of echocardiographic structural changes in the right heart, particularly when assessing patients with poor cardiac function at the initial diagnosis.

In addition to echocardiography and NYHA-FC, the other variables incorporated into the GENTH score model have also been extensively reported. Hematocrit, in particular, has been found to hold more clinical significance than hemoglobin (5) and serves as a valuable indicator for evaluating pulmonary vascular resistance (PVR) in high-altitude regions (52). Moreover, erythrocytosis and hepatic congestion, which reflect elevated blood volume and right heart dysfunction, often result in increased bilirubin levels (39). Additionally, previous studies have suggested that long-term residence in high-altitude regions contributes to increased glycated hemoglobin, which may be linked to hypoxia-inducible factor activation (36) and alterations in gluconeogenesis and glycogenesis influenced by activated neutrophils under altitude-induced hypoxia (53). Therefore, the GENTH score model effectively encapsulates the diverse changes in HAPH patients and proves to be a valuable tool for screening.

In our previous study, we used CTA to predict mPAP_RHC_, which improved the convenience of evaluating HAPH. However, CTA involves the use of a contrast agent, which belongs to large-scale examination (24). The current study refines the process for diagnosing and staging HAPH, improving screening effectiveness. We selected the cutoff value from the validation set with RHC as the standard for stratification, and further evaluation was guided by the GENTH score model. Specifically, we recommended more comprehensive echocardiography for potential HAPH patients with a GENTH score ranging from 18 to 30 points. Subsequently, the decision for RHC or follow-up should be based on the detailed echocardiographic report. RHC and intervention were strongly recommended for patients with a high suspicion of severe HAPH, indicated by a GENTH score of >30 points (Central Illustration). It is worthing mention that as the average altitude of the Shigatse area in Tibet is above 4,000 m, there was no influence of altitude variation and low altitude exposure. However, it has been reported that lower altitude exposure impacted highlanders and high-altitude hypoxia, thus the application of the GENTH scoring system may be limited in hospitals at lower altitudes (54).

**Central Illustration:**
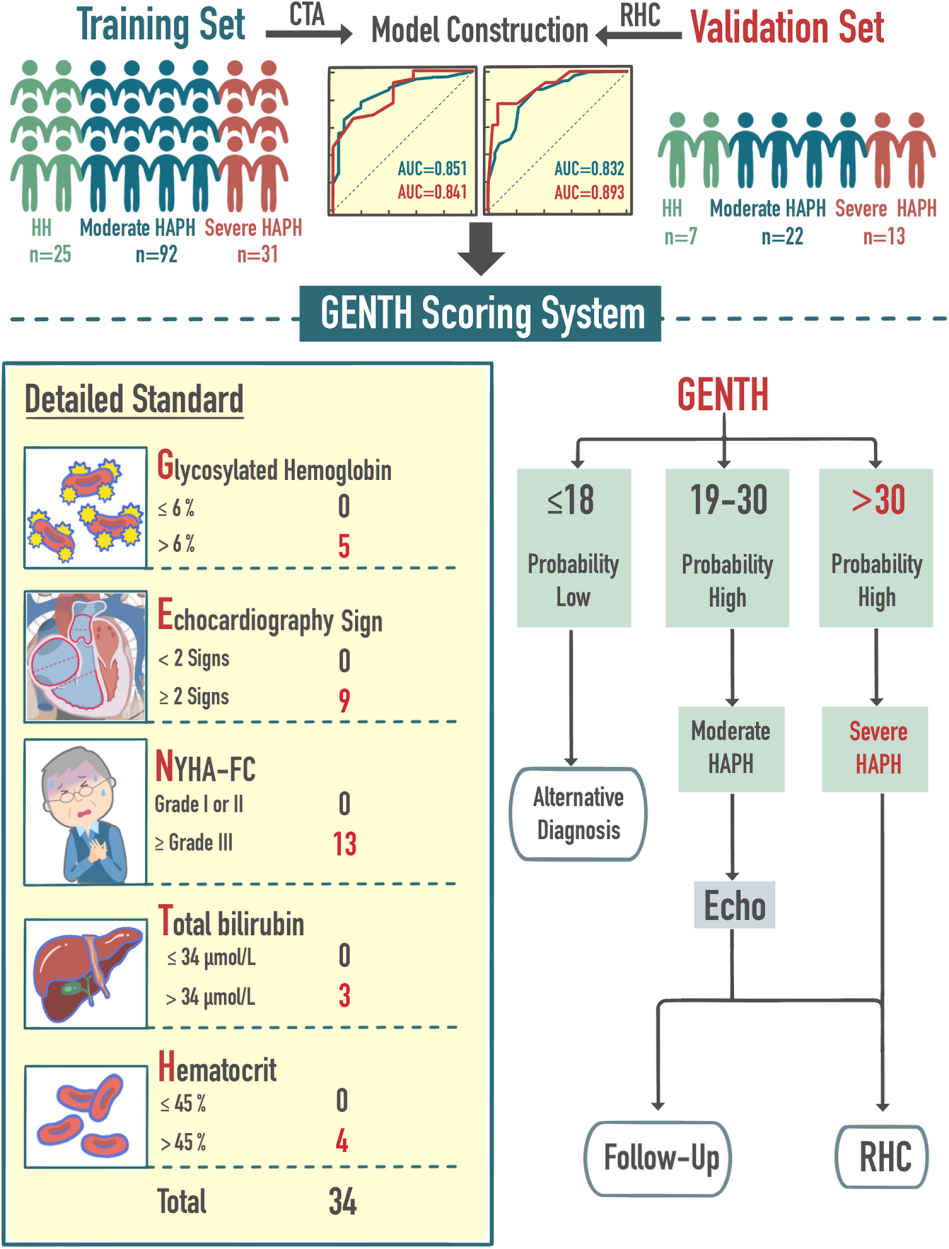
Brief model construction process and clinical application. The GENTH score was based on five risk factors: NYHA-FC, hematocrit, glycated hemoglobin, echocardiography sign, and total bilirubin. The GENTH score assisted the initial diagnosis and severity of HAPH and further guided the subsequent diagnosis and follow-up.

This study also had limitations. The development of clinical scoring standards requires an adequate sample size, which was challenging to achieve in our case. RHC data was lacking in the training group due to the difficulties associated with performing this procedure in high-altitude regions with limited medical resources. In addition, the presence of right heart dysfunction in HAPH patients (3) significantly impacts the accuracy of mPAP evaluation via echocardiography (11). Therefore, we employed a combined approach using limited RHC data and CTA to construct a predictive model for HAPH patients in the Shigatse People's Hospital (24), which was further applied to this retrospective large sample dataset for calculation and grouping. To further validate the accuracy of the GENTH scoring criteria, we conducted a prospective validation cohort that included RHC and pulmonary angiography, partially compensating for the limited sample size. Furthermore, lung function was not routinely performed in this research, so the exclusion of chronic obstructive pulmonary disease (COPD) may have been partially missed. The echocardiography only reported the parameters of the heart chamber and valvular regurgitation and did not assess the complete right heart function. More complete echocardiography parameters may be included to improve the accuracy of the model in the future. It is important to recognize that this study focused on an Asian population, and it is unclear whether the GENTH scoring model can be applied to other populations. However, previous studies in the Aksay and Andean highlanders have reported similar risk factors (4, 18). Thus, the GENTH scoring model may have predictive value in other high-altitude regions, but the relative weighted point value of these risk factors may differ. We look forward to its prediction results in other high-altitude regions.

## Conclusion

In this study, we introduced the GENTH score model, the first clinical prediction scoring system designed for both HAPH diagnosis and severity classification. The reliability and effectiveness of this scoring system were rigorously validated in a prospective cohort that employed RHC as the gold standard for diagnosing HAPH. The scoring system provided essential insights into the clinical characteristics associated with various disease stages, effectively addressing the limitations of echocardiography and ultimately improving the accuracy and efficiency of both HAPH diagnosis and severity classification in high-altitude regions.

## Data Availability

The raw data supporting the conclusions of this article will be made available by the authors, without undue reservation.
