# Supplementary material for: A novel clinical prediction scoring system of high-altitude pulmonary hypertension
Source: Front Cardiovasc Med. 2024 Jan 8;10:1290895. doi: 10.3389/fcvm.2023.1290895 (PMC10801263; doi:10.3389/fcvm.2023.1290895)
Supplement: Supplementary file 1 [file Datasheet1.pdf]

**FIGURE LEGENDS**

Figure 1 Flow chart of patients included and excluded in the retrospective training set.

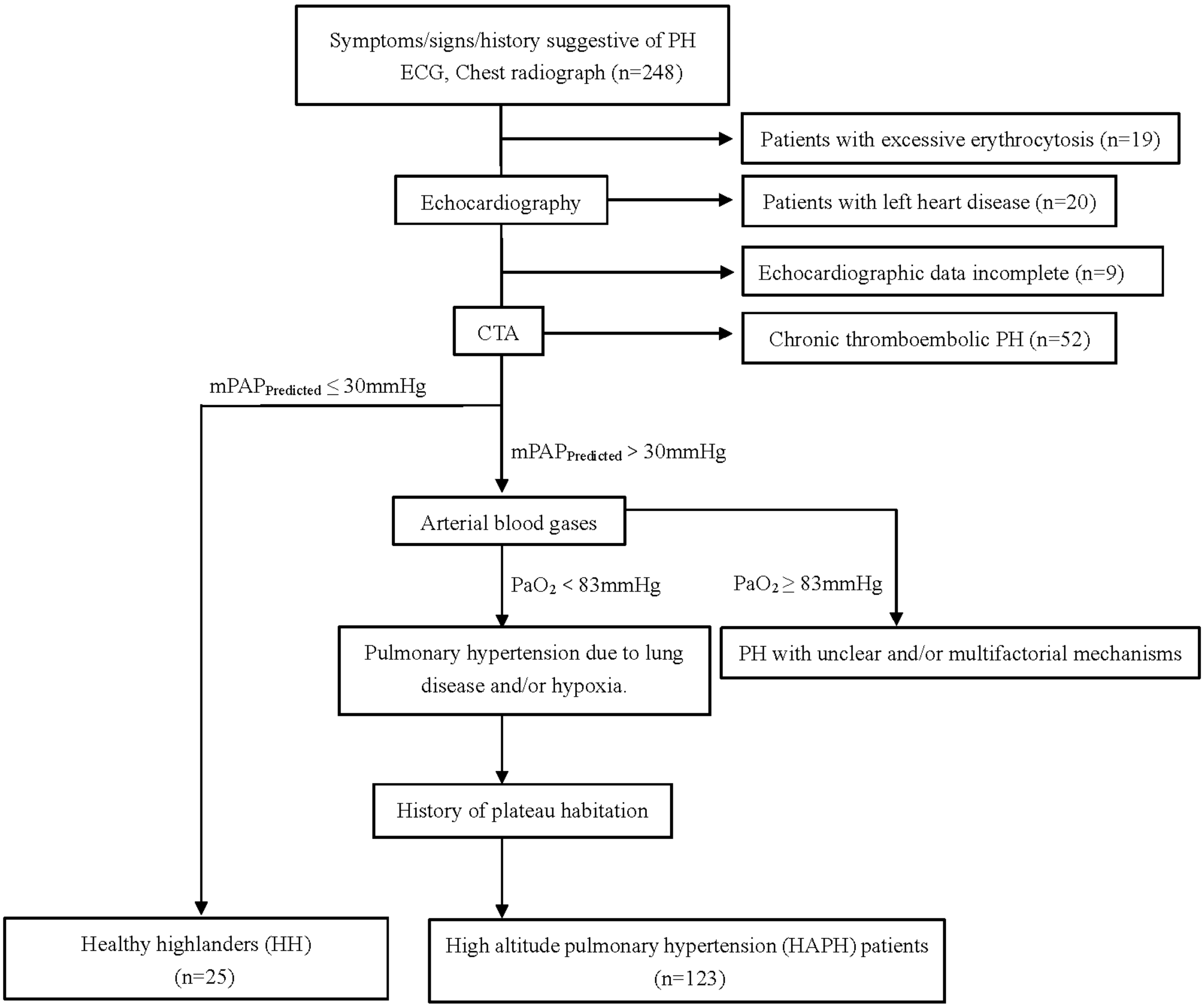


The inclusion criteria were the following: 1) individuals of both male and female aged 18 to 85; 2) admission to Shigatse People's Hospital between August 2020 and August 2022; 3) a documented history of residing in high-altitude areas for more than 20 years; 4) initial diagnosis suggesting PH; 5) performance of CTA during hospitalization.

Figure 2 Flow chart of patients included and excluded in the prospective validation set.

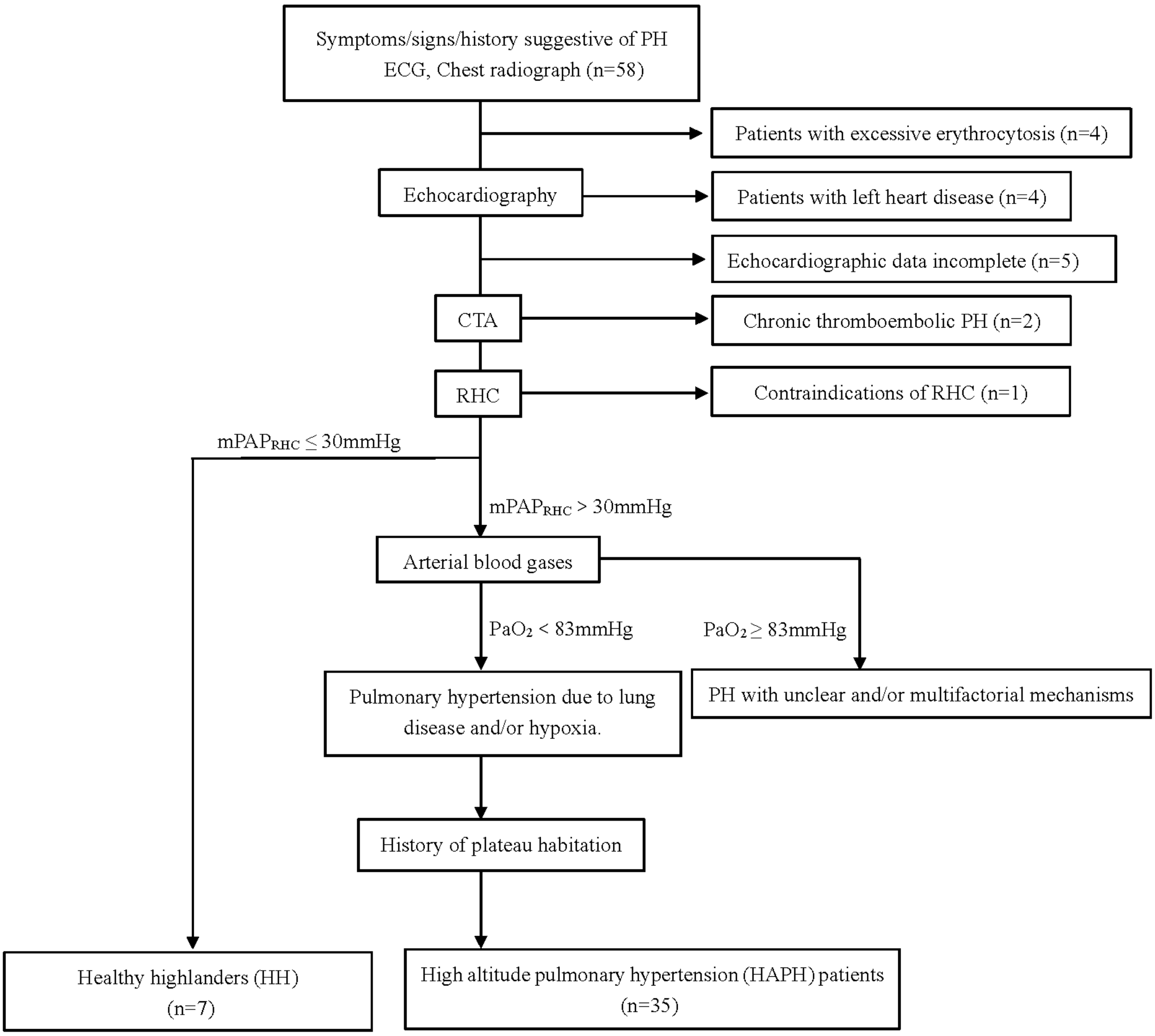


The inclusion criteria were the following: 1) individuals of both male and female aged 18 to 85; 2) admission to Shigatse People's Hospital between August 2022 and August 2023; 3) a documented history of residing in high-altitude areas for more than 20 years; 4) initial diagnosis suggesting PH; 5) performance of CTA during hospitalization; 5) no contraindications for undergoing RHC; 6) provision of informed consent.

**Table 1. Other baseline characteristics of individuals in the retrospective group**

| Characteristic | Total | 3-level of predicted mPAP | | | *p* value | *p* for trend |
|---|---|---|---|---|---|---|
| | | ≤30 (n=25) | 30-60 (n=92) | >60 (n=31) | | |
| Smoking Yes (%) | 25 (16.9) | 8 (32.0) | 11 (44.0) | 6 (24) | 0.055 | 0.283 |
| HR, bpm | 90 (76, 100) | 95 (89, 104) | 88 (76, 100) | 89 (76, 100) | 0.084 | 0.151 |
| SBP, mmHg | 120 (108, 144) | 115 (102, 131) | 125 (112, 145) | 116 (107, 137) | 0.158 | 0.932 |
| DBP, mmHg | 83 ± 16.3 | 81 ± 19.5 | 82 ± 15.8 | 84 ± 15.4 | 0.736 | 0.435 |
| Weight, kg | 63 (56, 67) | 60 (54, 65) | 63 (57, 68) | 62 (56, 69) | 0.313 | 0.475 |
| Height, cm | 164 (160, 169) | 165 (160, 169) | 164 (160, 168) | 165 (159, 170) | 0.589 | 0.968 |
| BMI, $kg/m^2$ | 23 (21, 25) | 21 (21, 24) | 23 (21, 25) | 23 (22, 25) | 0.141 | 1.000 |
| BSA, $m^2$ | 1.79 (1.68, 1.85) | 1.77 (1.70, 1.83) | 1.80 (1.70, 1.85) | 1.81 (1.67, 1.88) | 0.577 | 0.598 |
| $VO_2$, ml/min | 320 ± 25 | 319 ± 25 | 320 ± 26 | 321 ± 22 | 0.904 | 0.672 |
| CRP, mg/l | 10 (3, 44) | 14 (4, 99) | 10 (3, 43) | 9 (3, 43) | 0.176 | 0.002[b] |
| WBC, $\times 10^9$/l | 6.0 (4.7, 8.0) | 5.6 (4.9, 12.1) | 6.0 (4.9, 8.3) | 6.0 (4.3, 6.8) | 0.243 | 0.249 |
| Neu, % | 71 (63, 78) | 75 (65, 82) | 70 (63, 78) | 67 (59, 74) | 0.071 | 0.045[b] |
| FIB, g/l | 3.1 (2.1, 4.6) | 3.6 (2.1, 4.6) | 3.0 (2.0, 4.4) | 3.0 (2.0, 4.8) | 0.977 | 0.712 |
| D-D dimer, mg/l | 1.8 (1.0, 3.3) | 3.5 (1.1, 4.9) | 1.6 (0.8, 3.0) | 2.0 (1.2, 3.0) | 0.088 | 0.048[b] |
| PH | 7.41 (7.00, 7.46) | 7.40 (7.23, 7.47) | 7.41 (7.00, 7.47) | 7.41 (7.00, 7.44) | 0.558 | 0.323 |
| $SaO_2$, % | 81 (74, 90) | 78 (76, 92) | 80 (74, 89) | 83 (71, 90) | 0.774 | 0.362 |
| HDL-C, mmol/l | 1.03 (0.86, 1.19) | 1.09 (0.75, 1.20) | 1.05 (0.95, 1.19) | 0.86 (0.80, 1.18) | 0.127 | 0.379 |
| LDL-C, mmol/l | 1.77 (1.39, 2.00) | 1.76 (1.38, 1.95) | 1.84 (1.44, 2.08) | 1.66 (1.30, 2.00) | 0.252 | 0.932 |
| CK-MB, ng/ml | 2.46 (1.45, 5.30) | 1.45 (1.00, 5.74) | 2.42 (1.71, 4.88) | 2.80 (1.55, 5.57) | 0.139 | 0.285 |
| Myoglobin, ng/ml | 78 (38, 118) | 72 (33, 88) | 70 (43, 111) | 118 (36, 163) | 0.286 | 0.361 |
| cTnT, ng/ml | 0.38 (0.20, 0.65) | 0.49 (0.13, 0.65) | 0.38 (0.23, 0.65) | 0.38 (0.16, 0.65) | 0.794 | 0.923 |
| NT-proBNP, pg/l | 1,239 (192, 3,555) | 442 (134, 2,009) | 1,239 (178, 3,697) | 2,584 (243, 3,967) | 0.079 | 0.150 |

Data are mean ± SD or median ($P_{25}$, $P_{75}$). Group differences were assessed by one-way ANOVA, chi-square test or Kruskal-Wallis H tests. BNP = B-Type natriuretic peptides; CRP = C-reactive protein; FIB = fibrinogen; HDL-C = high-density lipoprotein; Neu% = neutrophilic granulocyte percentage; $PaCO_2$ = carbon dioxide partial pressure; $PaO_2$ = oxygen partial pressure. [a]$p$ <0.05: The group difference assessed by the Kruskal-Wallis H test or chi-square test was significant; [b]$p$ <0.05: The trend evaluated by linear regression analysis or Cochran-Armitage test was significant.

**Table 2 Estimated Difference (95% CI) in clinical laboratory indicators**

| Characteristic | Estimated Difference (95%CI) | | | *p* value |
|---|---|---|---|---|
| | 30-60 vs. ≤30 mmHg | ≤30 vs. >60 mmHg | 30-60 vs. >60 mmHg | |
| CRP, mg/l | 4.3 (-2.7-37.5) | 4.0 (-1.0-12.7) | 7.1 (-0.8-50.8) | 0.176 |
| WBC, $\times 10^9$/l | 0.4 (-0.9-1.8) | 0.7 (-0.2-1.6) | 1.1 (-0.4-3.0) | 0.243 |
| Neu, % | 4.7 (-1.5-10.5) | 3.4 (-1.9-9.1) | 8.0 (1.1-15.4) [b] | 0.071 |
| RBC, $\times 10^{12}$/l | 0.36 (-0.1-0.8) | 0.43 (-0.06-0.84) | 0.73 (0.14-1.43) [b] | 0.038[a] |
| HGB, g/l | 16 (1-31) [b] | 13 (0-26) | 28 (12-48) [b] | 0.005[a] |
| Hematocrit, % | 5.2 (0.6-9.0) [b] | 5.5 (2.1-9.6) [b] | 10.6 (5.5-15.5) [b] | <0.001[a] |
| ALT, U/l | 10 (-2-21) | 6 (-4-20) | 3 (-14-18) | 0.209 |
| AST, U/l | 10 (1-27) [b] | 11 (1-23) [b] | 1 (-18-18) | 0.028[a] |
| Albumin, g/l | 2.3 (-0.3-4.9) | 1.3 (-1.1-3.8) | 3.7 (0.5-6.7) [b] | 0.086 |
| Total bilirubin, μmol/l | 2.7 (-2.8-7.3) | 13.3 (6.3-21.1) [b] | 10.9 (2.3-20.9) [b] | <0.001[a] |
| CREA, μmol/l | 4.0 (-8.0-16.8) | 9.1 (-2.8-21.1) | 3.1 (-9.0-17.7) | 0.235 |
| UA, μmol/l | 27 (-49-93) | 106 (38-183) [b] | 125 (36-229) [b] | 0.006[a] |
| BUN, mmol/l | 0.5 (-0.3-1.5) | 1.0 (-0.0-2.3) | 0.5 (-0.8-2.1) | 0.094 |
| NT-proBNP, pg/l | 315 (-56-1434) | 270 (-211-1280) | 1116 (117-2516) [b] | 0.079 |
| PT, s | 0.4 (-0.7-1.4) | 1.2 (0.1-2.4) [b] | 1.9 (0.5-3.2) [b] | 0.027[a] |
| FIB, g/l | 0.1 (-0.6-0.8) | 0.0 (-0.74-0.65) | 0.0 (-1.2-0.9) | 0.977 |
| D-D dimer, mg/l | 1.05 (0.03-2.31) [b] | 0.27 (-0.30-0.75) | 1.00 (-0.35-2.24) | 0.088 |
| PH | 0.03 (-0.3-0.10) | 0.00 (-0.05-0.04) | 0.02 (-0.03-0.09) | 0.558 |
| $PaO_2$, mmHg | 1.8 (-4.8-8.0) | 0.24 (-5.0-5.0) | 2.0 (-0.60-10.0) | 0.822 |
| $PaCO_2$, mmHg | 3.5 (-0.1-7.5) | 2.5 (-1.9-6.7) | 6.1 (1.7-11.8) [b] | 0.046[a] |
| BE, mmol/l | 2 (0-4) [b] | 1 (-1-3) | 3 (1-6) [b] | 0.036[a] |
| SaO2, % | 1.9 (-3.2-6.0) | 1.0 (-4.0-6.0) | 0.0 (-6.0-8.0) | 0.774 |
| HCO3-, mmol/l | 1.8 (0.3-4.0) | 0.7 (-1.6-2.9) | 2.3 (-0.2-5.6) | 0.157 |
| TC, mmol/l | 0.0 (-0.3-0.3) | 0.5 (0.1-0.7) [b] | 0.5 (-0.1-0.8) | 0.048[a] |
| TG, mmol/l | 0.07 (0.02-0.18) [b] | 0.06 (-0.05-0.16) | 0.16 (0.02-0.29) [b] | 0.025[a] |
| HDL-C, mmol/l | 0.03 (-0.09-0.19) | 0.14 (0.01-0.23) [b] | 0.08 (-0.13-0.27) | 0.127 |
| LDL-C, mmol/l | 0.1 (-0.1-0.3) | 0.16 (-0.05-0.39) | 0.04 (-0.23-0.31) | 0.252 |
| Blood glucose, mmol/l | 0.3 (-0.1-0.6) | 0.2 (-0.1-0.5) | 0.43 (-0.02-0.83) | 0.120 |
| CK-MB, ng/ml | 0.5 (-0.3-1.3) | 0.6 (-0.2-1.7) | 1.0 (-0.1-2.3) | 0.139 |
| Myoglobin, ng/ml | 5.4 (-13.6-19.3) | 15.7 (-5.1-51.5) | 14.5 (-9.5-71.3) | 0.286 |
| cTnT, ng/ml | 0.0 (-0.1-0.2) | 0.0 (-0.1-0.1) | 0.1 (-0.1-0.2) | 0.794 |
| Glycated hemoglobin % | 0.15 (-0.01-0.49) | 0.40 (-0.20-0.68) [b] | 0.69 (0.41-0.79) [b] | <0.001[a] |

The 95% confidence interval for the difference between normally distributed data, pseudo-median and were calculated with Bonferroni estimate, Hodges-Lehmann estimate, respectively. ALT = alanine aminotransferase; AST = aspartate aminotransferase; BE = base excess; BNP = B-Type natriuretic peptides; BUN = Blood urea nitrogen; CK-MB = creatine phosphokinase-MB; CREA = creatinine; CRP = C-reactive protein; FIB = fibrinogen; $HCO_3^-$ = bicarbonate concentration; HDL-C = high-density lipoprotein; HGB = hemoglobin; LDL-C = low-density lipoprotein; Neu% = neutrophilic granulocyte percentage; $PaCO_2$ = carbon dioxide partial pressure; $PaO_2$ = oxygen partial pressure; PT = prothrombin time; RBC = erythrocyte; $SaO_2$ = oxyhemoglobin saturation; TC = total cholesterol; TG = triglyceride; UA = uric acid; WBC = leukocyte. [a]$p$ <0.05: The group difference assessed by chi-square test or Kruskal-Wallis H test was significant;

[b]$p$ <0.05: The estimated difference (95%CI) between the two groups was significant.

**Table 3 Estimated Difference (95% CI) of variables in echocardiography**

| Characteristic | Estimated Difference (95% CI) | | | *p* value |
|---|---|---|---|---|
| | 30-60 vs. ≤30 mmHg | ≤30 vs. >60 mmHg | 30-60 vs. >60 mmHg | |
| **Variables in echocardiography** | | | | |
| RAD1 enlarge (%) | 39 (17-57) [b] | 1 (-15-20) | 38 (12-58) [b] | 0.001[a] |
| RAD2 enlarge (%) | 41 (19-57) [b] | 12 (-7-27) | 53 (27-70) [b] | <0.001[a] |
| RVD1 enlarge (%) | 41 (19-57) [b] | 15 (-4-30) | 57 (31-73) [b] | <0.001[a] |
| RVD2 enlarge (%) | 24 (7-35) [b] | 10 (-8-30) | 35 (13-52) [b] | 0.011[a] |
| LAD1 enlarge (%) | 27 (5-44) [b] | 10 (-9-29) | 16 (-9-39) | 0.054 |
| LAD2 enlarge (%) | 19 (0-31) [b] | 1 (-18-17) | 18 (-3-36) | 0.128 |
| LVEDD enlarge (%) | 4 (-14-14) | 11 (-3-29) | 15 (-6-33) | 0.221 |
| LVESD enlarge (%) | 5 (-14-17) | 5 (-9-24) | 11 (-11-30) | 0.584 |
| MPAD enlarge (%) | 48 (27-63) [b] | 6 (-14-21) | 54 (28-71) [b] | <0.001[a] |
| Sign of right heart dysfunction ≥ 2 | 48 (26-63) [b] | 6 (-13-21) | 53 (27-70) [b] | <0.001[a] |
| TRPG, mmHg | 11 (2-21) [b] | 1 (-6-11) | 13 (2-25) [b] | 0.009[a] |
| PASP, mmHg | 10 (3-17) [b] | 0 (-7-10) | 10 (3-20) [b] | 0.009[a] |
| LVEF, % | 0 (-3-4) | 2 (-1-6) | 2 (-2-10) | 0.542 |

The 95% confidence interval for the difference between pseudo-median and independent proportions were calculated with Hodges-Lehmann estimate and Wilson procedure, respectively. LAD1 = transverse diameter of left atrium; LAD2 = vertical diameter of left atrium; LVEDD = left ventricle end-diastolic diameter; LVEF = left ventricular ejection fraction. LVESD = left ventricle end-systolic diameter; MPAD = main pulmonary artery diameter measured by echocardiography; PASP = echocardiographic pulmonary arterial systolic pressure estimate; RAD1 = transverse diameter of right atrium; RAD2 = vertical diameter of right atrium; rPA = the ratio of MPAD to aorta diameter; rRLA = the ratio of right to left atrial diameter; rSIVC = the ratio of superior to inferior vena cava diameter; RVD1 = transverse diameter of right ventricle; RVD2 = vertical diameter of right ventricle; TRPG = tricuspid regurgitation differential pressure. [a]$p$ <0.05: The group difference assessed by one-way ANOVA, Fisher's exact test or Kruskal-Wallis H test was significant; [b]$p$ <0.05: The estimated difference (95% CI) between the two groups was significant.

**Table 4 Clinical characteristics of patients on hospital admission in the training set**

| Parameter | HH vs HAPH | | | Moderate vs Severe HAPH | | |
|---|---|---|---|---|---|---|
| | HH (n=25) | HAPH (n=123) | *p* value | Moderate HAPH (n=92) | Severe HAPH (n=31) | *p* value |
| NYHA-FC | | | | | | |
| Grade I or II | 15 (60) | 38 (31) | | 36 (39) | 2 (6) | |
| ≥ Grade III | 10 (40) | 85 (69) | 0.006[a] | 56 (61) | 29 (94) | 0.001[a] |
| Hematocrit % | | | | | | |
| ≤ 45 | 21 (84) | 61 (50) | | 53 (58) | 8 (26) | |
| > 45 | 4 (16) | 62 (50) | 0.002[a] | 39 (42) | 23 (74) | 0.002[a] |
| Total bilirubin, µmol/l | | | | | | |
| ≤ 34 | 22 (88) | 67 (55) | | 57 (62) | 10 (15) | |
| > 34 | 3 (12) | 56 (45) | 0.002[a] | 35 (38) | 21 (68) | 0.004[a] |
| Uric acid, µmol/l | | | | | | |
| ≤ 420 | 19 (76) | 81 (66) | | 58 (63) | 12 (39) | |
| > 420 | 6 (24) | 42 (34) | 0.323 | 34 (37) | 19 (61) | 0.018[a] |
| Prothrombin time, s | | | | | | |
| ≤ 14 | 21 (84) | 92 (75) | | 26 (28) | 7 (23) | |
| > 14 | 4 (16) | 31 (25) | 0.324 | 66 (72) | 24 (77) | 0.537 |
| Glycated hemoglobin, % | | | | | | |
| ≤ 6.0 | 19 (76) | 81 (66) | | 76 (83) | 16 (52) | |
| > 6.0 | 6 (24) | 42 (34) | 0.323 | 16 (17) | 15 (48) | 0.001[a] |
| Echocardiography sign | | | | | | |
| < 2 | 19 (76) | 33 (27) | | 66 (72) | 15 (48) | |
| ≥ 2 | 6 (24) | 90 (73) | <0.001[a] | 26 (28) | 16 (52) | 0.018[a] |

NYHC-FC = New York Heart Association-functional class. [a]*p* <0.05: The group difference assessed by chi-square test or Fisher's exact test was significant

**Table 5 The univariate logistic regression of potential risk factors.**

| Parameter | HH vs. HAPH | | Moderate vs. Severe HAPH | |
|---|---|---|---|---|
| | OR (95% CI) | *p* value | OR (95% CI) | *p* value |
| NYHA-FC | | | | |
| Grade I or II | 1 | \ | 1 | \ |
| ≥ Grade III | 3.355 (1.382-8.145) | 0.007[a] | 9.321 (2.095-41.477) | 0.003[a] |
| Hematocrit % | | | | |
| ≤ 45 | 1 | \ | 1 | \ |
| > 45 | 5.336 (1.730-16.455) | 0.004[a] | 3.907 (1.581-9.653) | 0.003[a] |
| Total bilirubin, μmol/l | | | | |
| ≤ 34 | 1 | \ | 1 | \ |
| > 34 | 1.769 (0.563-5.554) | 0.328 | 4.453 (1.834-10.812) | 0.001[a] |
| Uric acid, μmol/l | | | | |
| ≤ 420 | 1 | \ | 1 | \ |
| > 420 | 1.642 (0.610-4.422) | 0.327 | 2.708 (1.171-6.259) | 0.020[a] |
| Prothrombin time, s | | | | |
| ≤ 14 | 1 | \ | 1 | \ |
| > 14 | 2.398 (0.896-6.419) | 0.082 | 2.701 (1.169-6.241) | 0.020[a] |
| Glycated hemoglobin, % | | | | |
| ≤ 6.0 | 1 | \ | 1 | \ |
| > 6.0 | 6.129 (1.743-21.552) | 0.005[a] | 3.42 (1.443-8.104) | 0.005[a] |
| Echocardiography sign | | | | |
| < 2 points | 1 | \ | 1 | \ |
| ≥ 2 points | 8.636 (3.175-23.494) | 0.001[a] | 1.351 (0.519-3.515) | 0.538 |

NYHC-FC = New York Heart Association-functional class. [a]$p < 0.05$: The group difference assessed by univariate logistic regression test was significant.

**Table 6 Characteristics of clinical laboratory indicators in the validation set.**

| Characteristic | Total | 3-level of $mPAP_{RHC}$ (mmHg) | | | *p* value | *p* for trend |
|---|---|---|---|---|---|---|
| | | ≤30 (n=7) | 30-60 (n=22) | >60 (n=13) | | |
| $mPAP_{Predicted}$ | 48 (35, 64) | 24 (20, 33) | 44 (38, 55) | 71 (62, 87) | <0.001[a] | <0.001[b] |
| PAWP | 10 (9, 12) | 11 (8, 12) | 10 (8, 11) | 11 (10, 12) | 0.332 | 0.757 |
| CRP, mg/l | 1.6 (0.5, 6.0) | 0.2 (0.2, 0.8) | 1.5 (0.5, 3.7) | 3.7 (1.5, 8.6) | 0.064 | 0.441 |
| WBC, $\times10^9$/l | 5.2 (4.4, 6.7) | 5.1 (4.4, 7.7) | 5.2 (4.3, 6.8) | 4.7 (4.4, 6.3) | 0.676 | 0.087 |
| Neu, % | 67 ± 10 | 66 ± 11 | 68 ± 10 | 65 ± 8 | 0.675 | 0.828 |
| RBC, $\times10^{12}$/l | 5.5 ± 1.0 | 5.2 ± 1.1 | 5.3 ± 1.0 | 5.9 ± 1.0 | 0.048[a] | 0.025[b] |
| HGB, g/l | 158 ± 32 | 162 ± 30 | 148 ± 31 | 175 ± 31 | 0.021[a] | 0.010[b] |
| Hematocrit, % | 49 (41, 58) | 44 (38,53) | 48 (42, 61) | 53 (50, 58) | 0.017[a] | 0.011[b] |
| ALT, U/l | 26 (16, 47) | 31 (21, 44) | 21 (14, 42) | 30 (19, 68) | 0.308 | 0.270 |
| AST, U/l | 28 (23, 53) | 25 (21, 51) | 27 (21, 41) | 50 (27, 79) | 0.093 | 0.072 |
| Albumin, g/l | 36 (32, 41) | 47 (40, 66) | 35 (33, 41) | 32 (28, 36) | 0.002[a] | 0.001[b] |
| Total bilirubin, μmol/l | 21 (16, 35) | 15 (13, 18) | 19 (16, 36) | 24 (20, 34) | 0.037[a] | 0.080 |
| CREA, μmol/l | 76 ± 21 | 75 ± 18 | 79 ± 20 | 72 ± 23 | 0.648 | 0.776 |
| UA, μmol/l | 362 (280, 426) | 260 (187, 383) | 324 (261, 408) | 419 (376, 578) | 0.005[a] | 0.002[b] |
| BUN, mmol/l | 4.6 (3.4, 6.1) | 4.6 (3.5, 6.1) | 4.9 (3.2, 6.1) | 4.5 (3.7, 6.0) | 0.908 | 0.477 |
| NT-proBNP, pg/l | 958 (353, 2,941) | 175 (75, 5,123) | 995 (443, 3,037) | 942 (482, 2,760) | 0.690 | 0.874 |
| PT, s | 13 (12, 14) | 11 (10, 13) | 13 (12, 14) | 13 (13, 14) | 0.085 | 0.145 |
| FIB, g/l | 2.0 (1.8, 2.6) | 2.5 (2.3, 2.8) | 2.0 (1.8, 2.4) | 1.9 (1.8, 2.8) | 0.275 | 0.351 |
| D-D dimer, mg/l | 0.6 (0.3, 1.4) | 0.3 (0.2, 0.5) | 0.7 (0.4, 1.7) | 0.8 (0.5, 1.1) | 0.077 | 0.508 |
| PH | 7.42 (7.39, 7.44) | 7.42 (7.40, 7.45) | 7.42 (7.40, 7.46) | 7.40 (7.37, 7.42) | 0.184 | 0.688 |
| PaO2, mmHg | 51 (48, 60) | 50 (47, 56) | 53 (48, 65) | 49 (40, 56) | 0.337 | 0.828 |
| PaCO2, mmHg | 31 (26, 35) | 32 (30, 32) | 31 (25, 38) | 31 (26, 36) | 0.974 | 0.886 |
| BE, mmol/l | -4.0 (-7.0, 0.0) | -3.7 (-6.0, -2.0) | -4.0 (-7.5, 2.0) | -5.0 (-8.5, -2.5) | 0.494 | 0.963 |
| SaO2, % | 84 (80, 90) | 84 (83, 86) | 86 (80, 92) | 82 (73, 89) | 0.579 | 0.338 |
| HCO3-, mmol/l | 21 (19, 24) | 21 (21, 22) | 20 (18, 26) | 21 (17, 23) | 0.621 | 0.521 |
| TC, mmol/l | 3.0 (2.5, 3.7) | 3.4 (3.2, 4.1) | 3.0 (2.5, 3.7) | 2.6 (1.9, 3.3) | 0.047[a] | 0.026[b] |
| TG, mmol/l | 0.81 (0.69, 1.09) | 1.07 (0.96, 1.13) | 0.80 (0.71, 1.07) | 0.64 (0.53, 1.05) | 0.064 | 0.163 |
| HDL-C, mmol/l | 0.96 (0.71, 1.17) | 1.19 (1.11, 1.30) | 1.00 (0.76, 1.17) | 0.64 (0.52, 1.05) | 0.016[a] | 0.017[b] |
| LDL-C, mmol/l | 1.61 (1.38, 2.09) | 2.02 (1.57, 2.13) | 1.60 (1.38, 2.18) | 1.56 (1.00, 2.04) | 0.181 | 0.084 |
| Blood glucose, mmol/l | 4.2 (3.8, 4.5) | 4.4 (3.9, 5.3) | 4.2 (3.9, 5.3) | 4.0 (3.5, 4.2) | 0.034[a] | 0.013[b] |
| CK-MB, ng/ml | 1.71 (1.49, 3.32) | 1.05 (0.56, 2.15) | 1.66 (1.49, 3.06) | 2.60 (1.50, 4.76) | 0.032[a] | 0.015[b] |
| Myoglobin, ng/ml | 35 (20, 53) | 53 (34, 53) | 30 (16, 49) | 33 (21, 66) | 0.204 | 0.561 |
| cTnT, ng/ml | 0.20 (0.01, 0.45) | 0.45 (0.23, 0.46) | 0.14 (0.01, 0.44) | 0.02 (0.01, 0.47) | 0.176 | 0.561 |
| Glycated hemoglobin % | 6.3 (5.6, 6.8) | 5.48 (4.10, 5.70) | 6.45 (5.70, 7.13) | 6.30 (5.64, 6.70) | 0.034[a] | 0.038[b] |

Data are mean ± SD or median ($P_{25}$, $P_{75}$). Group differences were assessed by one-way ANOVA or Kruskal-Wallis H tests. AST = aspartate aminotransferase; BE = base excess; BNP = B-Type natriuretic peptides; BUN = Blood urea nitrogen; CK-MB = creatine phosphokinase-MB; CREA = creatinine; CRP = C-reactive protein; FIB = fibrinogen; $HCO_3^-$ = bicarbonate concentration; HDL-C = high-density lipoprotein; HGB =

hemoglobin; LDL-C = low-density lipoprotein; Neu% = neutrophilic granulocyte percentage; $PaCO_2$ = carbon dioxide partial pressure; $PaO_2$ = oxygen partial pressure; PAWP = pulmonary artery wedge pressure; PT = prothrombin time; RBC = erythrocyte; $SaO_2$ = oxyhemoglobin saturation; TC = total cholesterol; TG = triglyceride; UA = uric acid; WBC = leukocyte. [a]$p < 0.05$: The group difference assessed by Kruskal-Wallis H test was significant; [b]$p < 0.05$: The linear association between continuous variables was significant, and the elevated $mPAP_{RHC}$ is evaluated by linear regression analysis.

**Table 7 Characteristics of parameters of echocardiography in the validation set.**

| Characteristic | Group | Total | 3-level of $mPAP_{RHC}$ (mmHg) | | | *p* value | *p* for trend |
|---|---|---|---|---|---|---|---|
| | | | ≤30 (n=7) | 30-60 (n=22) | >60 (n=13) | | |
| RAD1, mm | Enlarge (%) | 38 (90) | 5 (71) | 20 (91) | 13 (0) | 0.094 | 0.048[b] |
| RAD2, mm | Enlarge (%) | 39 (93) | 5 (71) | 21 (95) | 13 (0) | 0.092 | 0.033[b] |
| RVD1, mm | Enlarge (%) | 33 (79) | 3 (43) | 18 (82) | 12 (92) | 0.049[a] | 0.018[b] |
| RVD2, mm | Enlarge (%) | 37 (88) | 2 (29) | 22 (100) | 13 (100) | <0.001[a] | <0.001[b] |
| LAD1, mm | Enlarge (%) | 21 (50) | 2 (29) | 12 (45) | 7 (54) | 0.590 | 0.366 |
| LAD2, mm | Enlarge (%) | 25 (60) | 5 (71) | 12 (55) | 8 (62) | 0.766 | 0.793 |
| LVEDD, mm | Enlarge (%) | 32 (76) | 5 (71) | 15 (68) | 12 (92) | 0.268 | 0.198 |
| LVESD, mm | Enlarge (%) | 32 (76) | 5 (71) | 15 (68) | 12 (92) | 0.268 | 0.198 |
| MPAD, mm | Enlarge (%) | 31 (74) | 2 (29) | 16 (73) | 13 (100) | 0.002[a] | 0.001[b] |
| Sign of right heart dysfunction | 0 or 1 | 5 (12) | 4 (57) | 1 (5) | 0 (0) | | |
| | ≥ 2 | 37 (88) | 3 (43) | 21 (95) | 13 (100) | <0.001[a] | <0.001[b] |
| TRPG, mmHg | | 55 ± 25 | 36 ± 22 | 51 ± 19 | 72 ± 25 | 0.002[a] | 0.001[b] |
| PASP, mmHg | | 66 ± 26 | 46 ± 22 | 62 ± 20 | 83 ± 26 | 0.003[a] | 0.001[b] |
| LVEF, % | | 63 (58, 66) | 65 (41, 68) | 63 (53, 65) | 64 (60, 67) | 0.440 | 0.310 |

Data are median ($P_{25}$, $P_{75}$). The categorical variables are presented as absolute numbers (percentages). Group differences were assessed by Fisher's exact test or Kruskal-Wallis H tests. LAD1 = transverse diameter of left atrium; LAD2 = vertical diameter of left atrium; LVEDD = left ventricle end-diastolic diameter; LVEF = left ventricular ejection fraction. LVESD = left ventricle end-systolic diameter; MPAD = main pulmonary artery diameter measured by echocardiography; PASP = echocardiographic pulmonary arterial systolic pressure estimate; RAD1 = transverse diameter of right atrium; RAD2 = vertical diameter of right atrium; RVD1 = transverse diameter of right ventricle; RVD2 = vertical diameter of right ventricle; TRPG = tricuspid regurgitation differential pressure. [a]$p < 0.05$: The group difference assessed by Kruskal-Wallis H test or chi-square test was significant; [b]$p < 0.05$: The trend evaluated by linear regression analysis or Cochran-Armitage test was significant.

**Table 8. Spearman's correlation of $mPAP_{RHC}$ with $mPAP_{Predicted}$ and echocardiograph parameters**

| **Parameter** | **Avg±SD or M ($P_{25}$, $P_{75}$)** | **r** | ***p* value** |
|---|---|---|---|
| $mPAP_{RHC}$ | 52 (36, 66) | / | / |
| $mPAP_{Predicted}$ | 48 (35, 64) | 0.876 | <0.001[a] |
| Echocardiography sign | 3 (2, 3) | 0.529 | <0.001[a] |
| TRPG, mmHg | 55 ± 25 | 0.526 | <0.001[a] |
| PASP, mmHg | 66 ± 26 | 0.505 | 0.001[a] |

Data are median ($P_{25}$, $P_{75}$). mPAP = mean pulmonary arterial pressure; $mPAP_{Predicted}$ = mPAP predicted with CTA model; $mPAP_{RHC}$ = mPAP measured by right heart catheterization; PASP = echocardiographic pulmonary arterial systolic pressure estimate; TRPG = tricuspid regurgitation differential pressure. [a]$p<0.05$: The Spearman's correlation coefficient was significant, the correlation between independent variables and $mPAP_{RHC}$ was significant.
